# Anaerobic Degradation of the Plant Sugar Sulfoquinovose Concomitant With H_2_S Production: *Escherichia coli* K-12 and *Desulfovibrio* sp. Strain DF1 as Co-culture Model

**DOI:** 10.3389/fmicb.2018.02792

**Published:** 2018-11-27

**Authors:** Anna Burrichter, Karin Denger, Paolo Franchini, Thomas Huhn, Nicolai Müller, Dieter Spiteller, David Schleheck

**Affiliations:** ^1^Department of Biology, University of Konstanz, Konstanz, Germany; ^2^The Konstanz Research School Chemical Biology, University of Konstanz, Konstanz, Germany; ^3^Department of Chemistry, University of Konstanz, Konstanz, Germany

**Keywords:** anaerobic bacterial metabolism, sulfidogenesis, organosulfonate respiration, sulfoquinovosyldiacylglycerol, plant sulfolipid, biogeochemical carbon and sulfur cycle, gut microbiome, human health and disease

## Abstract

Sulfoquinovose (SQ, 6-deoxy-6-sulfoglucose) is produced by plants and other phototrophs and its biodegradation is a relevant component of the biogeochemical carbon and sulfur cycles. SQ is known to be degraded by aerobic bacterial consortia in two tiers via C_3_-organosulfonates as transient intermediates to CO_2_, water and sulfate. In this study, we present a first laboratory model for anaerobic degradation of SQ by bacterial consortia in two tiers to acetate and hydrogen sulfide (H_2_S). For the first tier, SQ-degrading *Escherichia coli* K-12 was used. It catalyzes the fermentation of SQ to 2,3-dihydroxypropane-1-sulfonate (DHPS), succinate, acetate and formate, thus, a novel type of mixed-acid fermentation. It employs the characterized SQ Embden-Meyerhof-Parnas pathway, as confirmed by mutational and proteomic analyses. For the second tier, a DHPS-degrading *Desulfovibrio* sp. isolate from anaerobic sewage sludge was used, strain DF1. It catalyzes another novel fermentation, of the DHPS to acetate and H_2_S. Its DHPS desulfonation pathway was identified by differential proteomics and demonstrated by heterologously produced enzymes: DHPS is oxidized via 3-sulfolactaldehyde to 3-sulfolactate (SL) by two NAD^+^-dependent dehydrogenases (DhpA, SlaB); the SL is cleaved by an SL sulfite-lyase known from aerobic bacteria (SuyAB) to pyruvate and sulfite. The pyruvate is oxidized to acetate, while the sulfite is used as electron acceptor in respiration and reduced to H_2_S. In conclusion, anaerobic sulfidogenic SQ degradation was demonstrated as a novel link in the biogeochemical sulfur cycle. SQ is also a constituent of the green-vegetable diet of herbivores and omnivores and H_2_S production in the intestinal microbiome has many recognized and potential contributions to human health and disease. Hence, it is important to examine bacterial SQ degradation also in the human intestinal microbiome, in relation to H_2_S production, dietary conditions and human health.

## Introduction

Sulfoquinovose (6-deoxy-6-sulfoglucose; SQ) is the polar headgroup of plant sulfolipids (sulfoquinovosyl diacylglycerols; SQDGs) in the photosynthetic (thylakoid) membranes of, essentially, all higher plants, ferns, mosses, algae as well as most phototrophic bacteria (Benson et al., 1959; Benson, 1963); SQ is also a component of the cell wall of archaea (Palmieri et al., 2013). SQ is one of the most abundant organosulfur species in the biosphere, with an estimated annual production of 10 billion tonnes and in a scale comparable with the amino acids cysteine and methionine (Harwood and Nicholls, 1979). Thus, SQ is an important component of the biogeochemical sulfur cycle and an understanding of its complete biodegradation, i.e., inclusive of a recycling of the organosulfur in form of inorganic sulfur species such as sulfate, is of importance.

**FIGURE 1:**
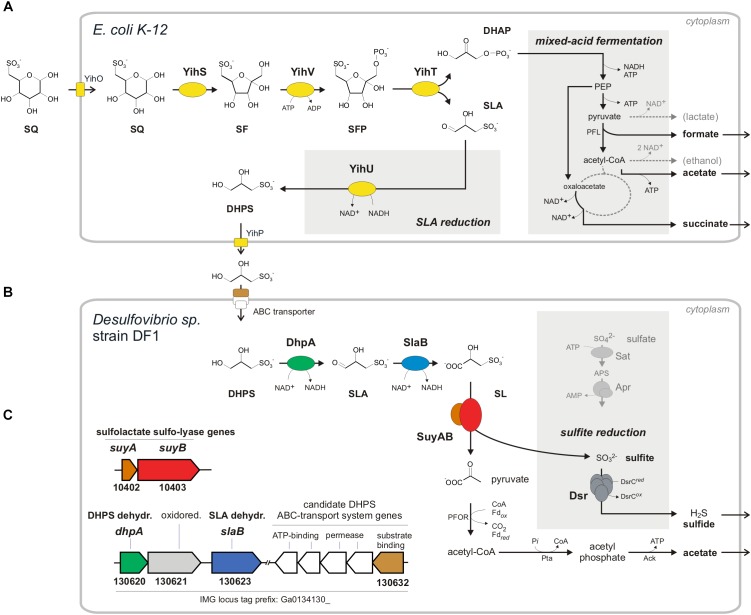
Anaerobic two-step degradation of SQ to H_2_S, as demonstrated in this study using a defined two-member bacterial co-culture. **(A)** Fermentation of sulfoquinovose (SQ) to 2,3-dihydoxypropane-1-sulfonate (DHPS), formate, acetate and succinate by *E. coli* K-12. SQ is metabolized and cleaved into DHAP and 3-sulfolactaldehyde (SLA) by a reaction sequence (enzymes YihS, V and T) analogous to the Embden-Meyerhof-Parnas pathway, as demonstrated previously for aerobic growth of *E. coli* (Denger et al., 2014). The C_3_-organosulfonate DHPS is excreted and available as substrate to other bacteria. Under anaerobic growth conditions, *E. coli* funnels most of the DHAP-carbon into mixed-acid fermentation (see schematic in the gray inset on the right) to succinate, formate and acetate as fermentation products. In addition, the reduction of the SLA to DHPS, as catalyzed by the previously characterized NADH-dependent SLA reductase YihU (Denger et al., 2014), serves as additional fermentation step (gray inset in the middle). **(B)** Fermentation of the DHPS to acetate and H_2_S by *Desulfovibrio* sp. strain DF1. As revealed in this study, DHPS is oxidized to 3-sulfolactate (SL) by two subsequent dehydrogenase reactions and the SL is cleaved into pyruvate and (bi)sulfite (${\mathrm{HSO}}_{3}^{-}$). The pyruvate is utilized for ATP generation concomitant with acetate excretion (and as carbon source for biomass formation, not shown). The sulfite is utilized as electron acceptor for sulfite respiration, as catalyzed by dissimilatory sulfite reductase (Dsr), and reduced to H_2_S (gray inset; for comparison, the ATP-consuming activation of sulfate is also shown). **(C)** The genes for DHPS-degradative enzymes identified by differential proteomics in *Desulfovibrio* sp. strain DF1 cells are indicated by the color coding **(B,C)** and/or by their IMG locus tag numbers; they are located on different contigs of the draft-genome sequence. In addition to the DHPS-desulfonation pathway genes, a candidate DHPS-transporter gene was identified by differential proteomics, i.e., for the soluble substrate binding protein (indicated in brown), which is co-encoded with candidate ABC-transporter permease and ATP-binding component genes (indicated in white), and a candidate aldehyde:ferredoxin oxidoreductase gene (oxidored.; IMG locus tag no. 130621) (see text). Other abbreviations used: SF, 6-deoxy-6-sulfofructose; SFP, 6-deoxy-6-sulfofructose phosphate; PEP, phosphoenolpyruvate; YihO, SQ importer; YihP, DHPS exporter; PFL, pyruvate-formate lyase; PFOR, pyruvate:ferredoxin oxidoreductase; Pta, phosphotransacetylase; Ack, acetate kinase; Sat, ATP sulfurylase; Apr, adenylyl-sulfate reductase.

Two bacterial pathways for degradation of SQ have been discovered in the recent years. The first is analogous to the Embden-Meyerhof-Parnas pathway of glycolysis and was found in *Escherichia coli* K-12 (Denger et al., 2014; Figure 1A).

Sulfoquinovose is transported into the cell by YihO, converted to 6-deoxy-6-sulfofructose (SF) by SQ isomerase YihS, phosphorylated to 6-deoxy-6-sulfofructose-1-phosphate (SFP) by kinase YihV and the SFP is cleaved by aldolase YihT. This results in one molecule of dihydroxyacetone phosphate (DHAP), which is used for energy generation and growth, and one molecule of 3-sulfolactaldehyde (SLA), which is not further degraded (not desulfonated) but reduced to 2,3-dihydroxypropane-1-sulfonate (DHPS) by NADH-dependent reductase YihU. The DHPS is then transported out of the cell most likely by YihP. All genes in *E. coli* required for this pathway are encoded in one gene cluster, which is a core feature of commensal and pathogenic *E. coli* species (Denger et al., 2014), together with an α-glucosidase gene (*yihQ*) for a hydrolysis of the SQ-glycoside (Speciale et al., 2016), an aldose epimerase gene (*yihR*) for an interconversion of the SQ epimers (Abayakoon et al., 2017) and an SQ-responsive DoeR-family repressor gene (*yihW/csqR*) (Shimada et al., 2018). This SQ Embden-Meyerhof-Parnas (SQ-EMP) pathway has been identified for aerobic growth of *E. coli* K-12 (Denger et al., 2014), but the potential of *E. coli* to utilize SQ also for anaerobic, fermentative growth has not yet been explored. This was the subject of the present study (see below). The second known pathway for a primary degradation of SQ (which is not a topic of this study) proceeds analogous to the Entner-Doudoroff pathway for glucose degradation and was studied in aerobic *Pseudomonas putida* (Felux et al., 2015). Here, also SLA is produced and not further degraded (not desulfonated), but the SLA is oxidized by an NAD^+^-dependent dehydrogenase to 3-sulfolactate (SL), rather than reduced to DHPS as with *E. coli* K-12, prior to excretion of the SL.

The two C_3_-organosulfonate intermediates DHPS or SL produced by primary degradation of SQ can be degraded completely by aerobic bacteria (Rein et al., 2005; Denger et al., 2006, 2009, 2012; Mayer et al., 2010) and were shown to play an important ecological role in ocean plankton (Durham et al., 2015; Landa et al., 2017). For a typical soil bacterium, *Cupriavidus pinatubonensis* JMP134, the first desulfonation pathway for DHPS has been described by Mayer et al. (2010). In the first steps, DHPS is oxidized via SLA to SL (Figure 1B) by DHPS dehydrogenase (HpsN). The SL is then cleaved by 3-sulfolactate sulfite-lyase (SuyAB) (EC 4.4.1.24) (Rein et al., 2005) into sulfite, which is excreted as sulfate (see below), and pyruvate, which is used for energy conservation and growth. Further, there are two other pathways for heterotrophic growth with SL known in aerobic bacteria. *Silicibacter pomeroyi* oxidizes SL to 3-sulfopyruvate by SL dehydrogenase (SlcD), followed by transamination of the 3-sulfopyruvate to cysteate (3-sulfoalanine) and desulfonation by deaminating cysteate sulfite-lyase (CuyA) (EC 4.4.1.25), yielding pyruvate, sulfite and ammonium (Denger et al., 2006). In *Roseovarius nubinhibens*, SL is oxidized also to 3-sulfopyruvate, but then decarboxylated to sulfoacetaldehyde and desulfonated by sulfoacetaldehyde acetyltransferase (Xsc) (EC 2.3.3.15), yielding acetyl phosphate and sulfite (Denger et al., 2009). Notably, these DHPS- or SL-utilizing aerobic bacteria (with exception of *S. pomeroyi*) employ sulfite dehydrogenases and, hence, oxidize the sulfite generated by the desulfonation reactions to sulfate.

All detailed examinations on the degradation of SQ have been performed under oxic conditions with aerobic bacteria. In contrast, degradation of SQ under anoxic conditions by anaerobic bacteria and its potential role as a novel substrate for hydrogen sulfide (H_2_S) production (sulfidogenesis), has not been explored. Clearly, SQ is available as a substrate also in anoxic habitats, for example from decomposition of plant matter in water-saturated soils and of macro- and micro-algae in marine and freshwater sediments, inside of photosynthetic microbial mats, but also in the gastrointestinal tracts of herbivorous and omnivorous animals and humans, for which SQ is part of their green-vegetable diet: the sulfolipid is found in significant amounts in many green vegetables, for example, in spinach with up to 36% of the total lipid content (Kuriyama et al., 2005).

Here, we demonstrate that anaerobic degradation of SQ concomitant with sulfide production is performed by a defined two-member consortium of laboratory model organisms: SQ-degrading *E. coli* K-12 and a DHPS-degrading *Desulfovibrio* sp. strain newly isolated from an anaerobic enrichment culture inoculated with sewage sludge. For the first degradation step, we established the growth physiology and a novel type of mixed-acid fermentation of *E. coli* and confirmed the involvement of the SQ-EMP pathway. For the second degradation step, we established the growth physiology of isolate *Desulfovibrio* sp. strain DF1 and a novel type of sulfidogenic organosulfonate fermentation for DHPS. Further, the key genes for the DHPS desulfonation pathway in the *Desulfovibrio* sp. strain were, firstly, identified by genome sequencing and proteomics and, secondly, confirmed in their functions using recombinant enzymes.

## Materials and Methods

### Chemicals

Racemic (*R*,*S*)-DHPS and (*R*,*S*)-SL were synthesized as described elsewhere (Wood, 1971; Roy et al., 2003; Mayer et al., 2010). SQ was provided by MCAT GmbH (Donaueschingen, Germany). Routine chemicals were from Merck, Roth or Sigma-Aldrich. NAD^+^ was purchased from Roche, IPTG from VWR and NADH from Biomol. PCR supplies were from New England BioLabs (PCR for cloning) and Genaxxon biosciences (colony PCR).

### Bacterial Strains

*Desulfovibrio* sp. strain DF1, which was enriched and isolated from anaerobic sewage sludge in this study, was deposited at the Leibniz Institute DSMZ-German Collection of Microorganism and Cell Cultures^1^ under the reference number DSM 107641. Its annotated draft genome can be accessed via integrated microbial genomes (IMG)^2^ under IMG project ID Gp0153975 (GOLD ID Ga0134130). *Escherichia coli* K-12 substrain MG1655 was available in the lab (Denger et al., 2014), as were the knockouts strains obtained previously from the *E. coli* Keio Knockout Collection (Baba et al., 2006).

### Growth Conditions

*Desulfovibrio* sp. strain DF1 and *E. coli* were grown anaerobically in a carbonate-buffered minimal medium reduced with 1 mM Titanuim(III)nitriloacetate (Ti(III)-NTA) (basal medium, Widdel and Pfennig, 1981; trace elements solution, Widdel et al., 1983; Ti(III)NTA solution, Moench and Zeikus, 1983; selenium-tungstate solution, Tschech and Pfennig, 1984; vitamin solution, Pfennig, 1978) and 12 mM SQ or 6 mM glucose (*E. coli*) or 10 mM DHPS (*Desulfovibrio* sp.). For sulfate-respiring growth conditions, *Desulfovibrio* sp. DF1 was grown with 10 mM lactate as the carbon source and electron donor and 20 mM sulfate as the terminal electron acceptor. The gas phase was 20% CO_2_ and 80% N_2_. All cultures were incubated at 30°C. Cultures for proteomic analysis, 2D-PAGE and enzyme assays in cell-free extracts were harvested in late exponential phase at an optical density (OD_580_) of about 0.4. The cells were resuspended in 50 mM Tris–HCl buffer, pH 8.0, containing 5 mM MgCl_2_, 1.7 μg/ml DNAse I and 1× Halt^TM^ protease inhibitor (ThermoFisher Scientific) and disrupted in a cooled French pressure cell.

*E. coli* for cloning and overexpression was grown in LB medium with, if necessary, 30 μg/ml kanamycin, 100 μg/ml ampicillin or 35 μg/ml chloramphenicol, aerobically on an orbital shaker at 37 or 15°C. For details on the cloning, see below.

### Enrichments and Isolation

Enrichment cultures (20 ml) were set up in 50 ml serum bottles with butyl rubber stoppers and N_2_/CO_2_ gas, and incubated at 30°C in the dark. The anoxic Ti(III)NTA-reduced medium described above was used, containing either 5 mM SQ as sole source of carbon and energy for fermentative growth or 5 mM SQ plus 20 mM sulfate as electron acceptor; 10 mM DHPS as sole substrate was provided later in the process of isolating *Desulfovibrio* sp. strain DF1. Initial inoculation was performed with 1 ml of anoxic material from a sewage plant, a biogas plant, garden compost or sulfidic sediment of Lake Constance, Germany. When growth of bacteria was detected by turbidity formation in the supernatant of the cultures and by microscopy (after 3 days to 2 weeks), about 10% (v/v) of the culture was transferred to fresh medium. After four to five transfers, pure cultures were obtained by repeated application of the agar shake dilution method (Pfennig, 1978).

### Draft-Genome Sequencing of *Desulfovibrio* sp. DF1, Assembly and Sequence Analysis

Genomic DNA was extracted from an overnight culture using JGI’s Bacterial Genomic DNA isolation protocol (JGI Bacterial DNA isolation CTAB-2012^3^). The whole-genome shotgun sequencing was performed by GATC Biotech (Konstanz, Germany) using the Illumina HiSeq2000 platform and a 125-bp paired-end library, which resulted in 15,158,760 reads (3.78 × 10^9^ total bases).

The trimming, mapping, as well as the *de novo* assembly of the raw reads, was performed at the Genomics Center of the University of Konstanz. The remaining adapters were removed and reads were trimmed by quality in CLC Genomics Workbench v6.5 (CLC bio, Aarhus, Denmark). To estimate the mapping rate against different *Desulfovibrio* genomes, the program Bowtie v2.2.3 (Langmead and Salzberg, 2012) was used to align the filtered reads against the genomes of *Desulfovibrio alcoholivorans* DSM 5433 (NCBI genome ID 32104), *Desulfovibrio desulfuricans* DSM 642 (ID 15717), *Desulfovibrio fairfieldensis* (ID 42991) and *Desulfovibrio fructosivorans* JJ (ID 2656). The highest mapping (62%) was against the genome of *D. alcoholivorans* and this genome was later use in the reference-guided scaffolding approach (see below). The reads were then assembled *de novo* using the program SOAPdenovo v2.04 (Luo et al., 2012) with a *k*-mer size of 67 and setting the minimum contig length at 200 bp. The scaffolding step of SOAPdenovo produced a final assembly including 340 sequences (N50: 141 Kb). The *de novo* assembled sequences were then scaffolded by the reference-guided algorithm implemented in Ragout 2.0b (Kolmogorov et al., 2014) using the genome of *D. alcoholivorans* as reference. The final assembly included 315 sequences (N50: 332 Mb). This assembly was uploaded to the DoE-JGI IMG annotation pipeline^4^. The size of the draft genome is 4.9 Mb with 4,333,987 DNA coding bases. The average G+C content is 64.68%. Analysis of genomes for orthologous gene clusters was done via the Gene Cassette Search and Neighborhood Regions Search options of the IMG/ER and IMG Human Microbiome Project (IMG/HMP) platforms.

### Gene Cloning

Each gene for heterologous expression was first amplified from genomic DNA by PCR using primers targeting the start codon or downstream of the stop codon, respectively, and that contained restriction sites for NdeI (CATATG) in front of the start codon or XhoI (CTCGAG) at the end of the reverse primer (followed each by three random bases needed for restriction efficiency). Primers were designed using DNAStar PrimerSelect, and SerialCloner for virtual PCR and ligation as a control tool. Primers were synthetised by Microsynth AG (Balgach, Switzerland). The primer sequences are provided in the Supplementary Table S2.

The PCR product was first cloned by blunt end cloning into a pJet suicide vector using the CloneJet PCR cloning kit (ThermoFisher Scientific). The plasmid was transformed into chemically competent *E. coli* NovaBlue cells, which were streaked onto a selective LB plates containing ampicillin; *E. coli* NovaBlue were chosen for improved transformation efficiency and plasmid stability. The resulting colonies were tested by colony PCR using the insert primers. Positive colonies were cultured in 5 ml LB with ampicillin and the plasmids were extracted using the Zyppy Plasmid Miniprep kit (Zymo Research). The plasmids were digested with NdeI and XhoI (New England BioLabs Inc.) simultaneously in CutSmart buffer for 2 h at 37°C. The digest was loaded onto an agarose gel, the insert bands were excised and extracted using the Gel extraction Mini Spin Column kit (Genaxxon biosciences).

As expression vector, we chose the pET 28a(+) vector (Novagen) that results in an N-terminally His-tagged protein, and which has restriction sites for NdeI and XhoI. The vector is designed for high expression levels from a T7 system under control of a lac repressor and carries a kanamycin resistance cassette. The empty vector was digested and gel-purified as described above. The NdeI and XhoI-digested, purified inserts were then ligated into the vector using T4 ligase; the resulting plasmid was again transformed into *E. coli* NovaBlue and streaked on selective plates containing kanamycin. Positive clones identified by colony PCR were picked into 5 ml LB medium with kanamycin, and after overnight growth, the plasmids were extracted and sequenced; primers for the T7 expression region were used for sequencing, as provided by GATC (Konstanz, Germany). GATCViewer and SerialCloner were used to confirm the correct insert sequences.

### Heterologous Expression and Protein Purification

For overexpression, the pET28 plasmid constructs were transformed into chemically competent *E. coli* Rosetta 2 DE3 cells; this strain enables expression under control of a T7 promoter and contains a rare codon usage plasmid (pRARE2) which also bears a chloramphenicol resistance. The transformed cells were cultured overnight in 5 or 10 ml of liquid LB medium containing kanamycin and chloramphenicol. For expression, 100 ml of LB with kanamycin and chloramphenicol were inoculated (2.5–5%) with the overnight culture and incubated shaking at 37°C until they reached an OD_580_ of about 0.5. Expression was induced by addition of 1 mM isopropyl-β-D-thiogalactopyranoside (IPTG). The cultures were incubated further either for 5–6 h at 37°C (for DHPS dehydrogenase) or at 15°C overnight in order to aid with folding and solubility (for SLA dehydrogenase, SuyA and SuyB).

Cells were harvested by centrifugation at 13,000 × *g* for 15 min and washed twice in 20 mM phosphate buffer, pH 7.2, containing 500 mM NaCl, 2.5 mM MgCl_2_, 10 U/ml DNaseI, 1× Halt^TM^ protease inhibitor (ThermoFisher Scientific) and 20 mM imidazole. Cells were disrupted by four passages through a French pressure cell, or by incubation with 5 mg/ml lysozyme for 1 h at 37°C followed by a freeze-thaw cycle and 5 min in an ultrasound bath. Cell debris was then removed by centrifugation at 17,000 × *g* for 10 min and the His-tagged protein was purified over a Ni-NTA column (His SpinTrap^TM^ kit, GE Healthcare) with wash steps at 20, 40 and 60 mM imidazole and elution at 500 mM imidazole. To remove the imidazole, the buffer was then exchanged to 50 mM Tris–HCl, pH 9.0, using Illustra^TM^ Nap^TM^-5 columns (GE Healthcare).

For anaerobic expression of the aldehyde:ferredoxin oxidoreductase gene, *E. coli* Rosetta 2 DE3 was grown anaerobically in the minimal medium described above containing also 0.4% glucose, 5 mM sodium nitrate, 0.1% yeast extract and the required antibiotics. IPTG was added anoxically to a final concentration of 1 mM when the cultures had reached an OD of about 0.5. The cells were incubated overnight at 30°C and harvested by centrifugation in the culture flasks at 1,200 × *g* for 30 min. The pellet was resuspended, transferred into a vial flushed with N_2_ gas and disrupted in a French pressure cell flushed with N_2_. The purification and buffer exchange were performed in an anoxic tent with an atmosphere of 95% N_2_ and 5% H_2_. All buffers were degassed under vacuum, flushed with N_2_, and additionally contained 3 mM of dithiothreitol.

### Enzyme Assays

Enzyme assays were conducted in 50 mM Tris–HCl buffer, pH 9.0. Assays with purified DHPS and SLA dehydrogenases, or with cell-free extracts, contained 5 mM DHPS and 10 mM NAD^+^. Typically, 0.8–1.0 μg/ml purified DHPS dehydrogenase and 16.5 μg/ml purified SLA dehydrogenase were used in an assay. Assays with cell-free extract were performed with 5 μl of extract per ml reaction (31.5 μg/ml total protein). Enzymes were heat inactivated at 100°C for 10 min in a heating block for negative control reactions.

The enzyme assay for the recombinant aldehyde:ferredoxin oxidoreductase was conducted in anoxic 50 mM Tris–HCl buffer, pH 7.4, under an atmosphere of 80% N_2_ and 20% CO_2_. The assay typically contained 5 μg of purified enzyme, 1 mM benzylviologen and/or NAD^+^ as electron acceptor and 0.5 mM lactaldehyde or acetaldehyde as substrate. Sulfolactaldehyde as substrate was produced under these anoxic conditions from 2 mM DHPS in the presence of 4 mM NAD^+^ and 1 mg of DHPS dehydrogenase. The reduction of benzylviologen was observed photometrically by measuring the absorption at 578 nm for 1 min after addition of the aldehyde substrate, and the reduction of NAD^+^ at 365 nm.

For the recombinant SuyAB enzyme, both subunits were expressed and purified separately. For the SL desulfonation assay, 0.15 mg/ml of SuyA and 0.6 mg/ml of SuyB were typically used (meaning equimolar amounts, with SuyB having approximately four times the mass of SuyA). The enzyme was first reconstituted from both subunits overnight at -20°C in 0.1 M MOPS buffer with 1 mM FeCl_2_ and 10% (v/v) glycerol. To prevent oxidation of Fe^2+^, the mixture was degassed under vacuum and saturated with 80% N_2_ and 20% CO_2_ gas before freezing. The reaction was started by addition of 2.5 mM (final concentration) SL. Samples for quantification of sulfite and pyruvate were taken discontinuously.

### Analytical Methods

For quantification of glucose, SQ, SL and DHPS, pyruvate, NADH and NAD^+^ a Shimadzu HPLC system, a hydrophilic interaction liquid chromatography column (Merck ZIC-HILIC, 5 μm, 200 Å, 150 mm × 2.1 mm) and a UV/Vis-DAD detector (Shimadzu SPD-M20A) and evaporative light scattering detector (ELSD) (Schambeck SFD ZAM3000) were used. The total eluent flow was 0.3 ml/min with acetonitrile as the eluent A and 0.1 M ammonium acetate plus 10% acetonitrile as eluent B. HPLC conditions were as follows: gradient from 90 to 75% A for 20 min; gradient from 75 to 65% for 10 min; isocratic at 65% A for 10 min; to 20% A within 1 min; wash step at 20% for 9 min; back to 90% A in 1 min; equilibration at 90% A for 19 min. Under these conditions, DHPS eluted at 13.5 min, glucose at 17.6 min, SL at 20.5 min, and SQ at 26.7 min (as detected by ELSD). Pyruvate eluted at 6.6 min, NADH at 26.6 min, and NAD^+^ at 28.6 min (as detected at 350 nm).

For qualitative detection of DHPS, SLA, and SL the same column and conditions were used on an Agilent 1100 HPLC system coupled to a LCQ ion trap mass spectrometer (ThermoFisher Scientific) for electrospray ionization tandem mass spectrometry (ESI-MS/MS) operated in the negative mode (ES-MS/MS). The compounds were detected using ion trace search of the [M-H]^-^ ions: *m/z* = 155 for DHPS, *m/z* = 153 for SLA, *m/z* = 169 for SL.

Short-chain fatty acids and alcohols against authentic standards were analyzed on a Shimadzu HPLC system with an Aminex column (Aminex HPX-87H, BioRad) and a refractive index detector (RID; Shimadzu RID-10A) at 60°C. The eluent was 5 mM H_2_SO_4_ at an isocratic flow of 0.6 ml/min. Under these conditions, the fermentation products succinate eluted at 11.7 min, formate at 14.0 min, acetate at 15.2 min and ethanol at 22.0 min; lactate eluted at 13.0 min and pyruvate at 20.0 min. This Aminex HPLC-RID method generally separates and detects carbohydrates, carboxylic acids, short-chain fatty acids, alcohols, ketones and other metabolites; no other products (peaks) than the ones stated in the Section “Results” were detected in any growth experiment (*E. coli* K-12 and *Desulfovibrio* sp. DF1).

Hydrogen production was tested with a Peak Perfomer 1 (Peak Laboratories) trace gas analyzing gas chromatography system with a reducing compound photometer detector; N_2_ was used as the carrier gas.

Sulfide in culture media was routinely measured using an adapted methylene blue assay with Na_2_S as standard (Cline, 1969). Therefore, 10 μl of sample was added immediately to 100 μl 0.1 M zinc acetate solution and the sample was stored at -20°C. For analysis, the solution was brought to 895 μl total volume by addition of MilliQ water and 100 μl of a 10 mM solution of *N*,*N*-dimethyl-*p*-phenylenediamine sulfate in 20% sulfuric acid was added. The reaction was started by addition of 5 μl of a solution of 5 g NH_4_Fe(SO_4_)_2_ in 2% sulfuric acid. After 45 min, the absorption was measured at 670 nm.

Total protein content of cell pellets was determined by a modified Lowry assay as described in ref. Kennedy and Fewson (1968), scaled to 1 ml total reaction volume. Protein concentration in solutions was determined by a the Bradford assay in 1-ml scale (Bradford, 1976).

Sulfite was measured after derivatization with *N*-(9-acridinyl)maleimide (NAM) (Akasaka et al., 1990). Briefly, 50 μl of fresh sample were added to 150 μl borate buffer (0.3 M boric acid, 0.3 M potassium chloride, adjusted to pH 9.3 with a solution of 0.3 M sodium carbonate and 0.02 M EDTA). 50 μl of a solution of 10 mM NAM dissolved in acetone were added and the derivatization reaction was incubated at 50°C for 30 min. The samples were then frozen to precipitate protein and centrifuged for 1 min at 14,500 × *g*. NAM-Sulfite was quantified using a Phenomenex Luna Omega column (5 μm PS C_18_ 100 Å, 150 mm × 3.0 mm) with a Phenomenex SecurityGuard guard cartridge kit and an UV detector under the following conditions: Solvent A, Milli-Q water with 0.1% (v/v) formic acid; solvent B, acetonitrile; total flow, 0.5 ml/min; column temperature, 30°C; detection wavelength, 254 nm. Gradient: 10% B for 3 min; gradient to 80% B in 10 min; reequilibration at 10% B for 7 min. The NAM-sulfite signal was split into two peaks at 3.4 and 4.0 min, respectively, and the peak areas were added up for quantification. A fresh solution of sodium sulfite was used as standard.

Pyruvate from SuyAB enzyme assays was measured as a 2,4-dinitrophenylhydrazine (DNPH) derivate. 50 μl of fresh sample were added to 50 μl of a solution of 0.5 mg/ml DNPH in acetonitrile with 0.1% phosphoric acid (Koivusalmi et al., 1999; Frey et al., 2018). The reaction was incubated for 2 h at room temperature. The samples were then frozen to precipitate protein and centrifuged for 1 min at 14,500 × *g*. DNPH-pyruvate was quantified on a Phenomenex Luna Omega column (5 μm PS C_18_ 100 Å, 150 mm × 3.0 mm) with a Phenomenex SecurityGuard guard cartridge kit and an UV detector under the following conditions. Solvent A, Milli-Q water with 0.1% (v/v) formic acid; solvent B, acetonitrile; total flow, 0.75 ml/min; column temperature, 40°C; detection wavelength, 360 nm. Gradient: 25% B for 4.5 min; to 70% B in 15 min; reequilibration at 25% B for 10.5 min. Pyruvate-DNPH eluted as a single peak at 12.3 min retention time and was quantified against freshly prepared standards using a freshly prepared sodium pyruvate stock solution.

### Proteomics

Cells were grown, harvested and disrupted by four passages through a French pressure cell as described above. Intact cells and cell debris was removed by centrifugation at 12,000 × *g* for 5 min, 4°C. The protein concentration in these cell-free extracts was determined by Bradford assay. The total protein was purified when samples of 200 μg of total protein were mixed with SDS-PAGE loading dye (Roti-Load 1, Carl Roth) and denatured at 100°C for 10 min. The samples were then loaded on an SDS gel (12% resolving gel, 6% stacking gel) and run at 30 mA for 1 h until the proteins had entered the stacking gel (without any separation). The gel was stained with Coomassie Blue (Meyer and Lamberts, 1965) and each total-protein band was excised and submitted to the Proteomics Facility at the University of Konstanz for identification by peptide fingerprinting-mass spectrometry (see below). For analyzing only the soluble proteins in cell-free extract directly (without any SDS-PAGE purification), the membrane fragments were removed first from the extract by ultracentrifugation at 104,000 × *g* for 35 min at 4°C, and then samples of the soluble protein were submitted to peptide fingerprinting-mass spectrometry.

The protein extracts, and the protein bands or spots excised from SDS-PAGE gels, were subjected to peptide fingerprinting-mass spectrometry (PF-MS) at the Proteomics Facility of the University of Konstanz^5^ as described previously (Schmidt et al., 2013; Denger et al., 2014; Felux et al., 2015) with the exception that each sample was analyzed twice on a Orbitrap Fusion with EASY-nLC 1200 (Thermo Fisher Scientific), and that Tandem mass spectra were searched against an appropriate protein database (retrieved from IMG) using Mascot (Matrix Science) and Proteome Discoverer V1.3 (Thermo Fisher Scientific) with “Trypsin” enzyme cleavage, static cysteine alkylation by chloroacetamide, and variable methionine oxidation.

### Protein Gel Electrophoresis

To evaluate the purity of recombinant proteins, aliquots of the protein preparations corresponding to 15 μg of total protein (Bradford assay) were mixed with 4× SDS-PAGE loading dye (Roti-Load 1, Carl Roth) and denatured at 100°C for 10 min. The samples were then loaded onto an SDS gel (12% acrylamide for the resolving gel, 4% for the stacking gel) and run at a constant voltage of 100 V; the gels were stained with Coomassie Blue.

For differential two-dimensional PAGE and identification of proteins spots by peptide fingerprinting-mass spectrometry (Denger et al., 2014; Felux et al., 2015), *Desulfovibrio* sp. DF1 was grown with 10 mM DHPS or with 15 mM lactate plus 30 mM of sulfate. The cells were grown and harvested as described above and resuspended in 50 mM Tris–HCl buffer, pH 8.1, containing 2.5 mM MgCl_2_, 10 U/ml DNaseI and 1× Halt protease inhibitor (ThermoFisher Scientific). The cells were disrupted by four passages through a French pressure cell, centrifuged for 5 min at 12,000 × *g* to remove cell debris and then for 30 min at 104,000 × *g* to obtain the soluble protein fraction. An amount corresponding to 500 μg of total protein was precipitated by adding five volumes of acetone followed by incubation overnight at -20°C. Acetone was removed after centrifugation at 9,000 × *g* for 20 min, the protein pellets were dried under air, and then resuspended in 8 M urea, 2 M thiourea, 3% CHAPS, 60 mM DTT and 0.2% Bio-Lyte 3/10 ampholytes (Bio-Rad); the solution was incubated for 30 min in a cooled ultrasonic bath, followed by 2 h of shaking at room temperature. The samples were then loaded each onto an IPG Bio-Rad ReadyStrip pH gradient strip (pH range 5–8) and incubated overnight to rehydrate the IPG strip. The isoelectric focusing was performed with a voltage ramp to 10,000 V in 3 h, and a total focusing of 40,000 Vh. Thereafter, the strip was equilibrated in equilibration buffer [6 M urea, 0.375 M Tris, 2% SDS, 20% glycerol 2% (w/v) DTT] for 10 min on an orbital shaker and then washed with the same buffer containing additionally 2.5% (w/v) iodoacetamide. The strip was then placed onto a 12% SDS PAGE gel and mounted with SDS gel buffer containing 0.5% agarose. The gel was run overnight at 40 mA and stained with Coomassie Blue. Protein spots of interest were excised and analyzed at the Proteomics Facility of University of Konstanz (see above).

## Results and Discussion

### *Escherichia coli* K-12 Employs the SQ Embden-Meyerhof-Parnas Pathway for a Mixed-Acid Fermentation of SQ

We cultivated *E. coli* K-12 MG1655 routinely under fermentative conditions with SQ in a carbonate-buffered mineral salts medium in the presence of Ti(III)NTA as reducing agent instead of Na_2_S, in order to allow for a co-cultivation of *Desulfovibrio* sp. and for a quantification of H_2_S production during DHPS degradation (see below). For the physiological growth experiments with *E. coli* K-12 in pure culture, we used mixed-acid fermentation of glucose, as well as its aerobic growth with SQ or glucose (Denger et al., 2014), as references.

**FIGURE 2:**
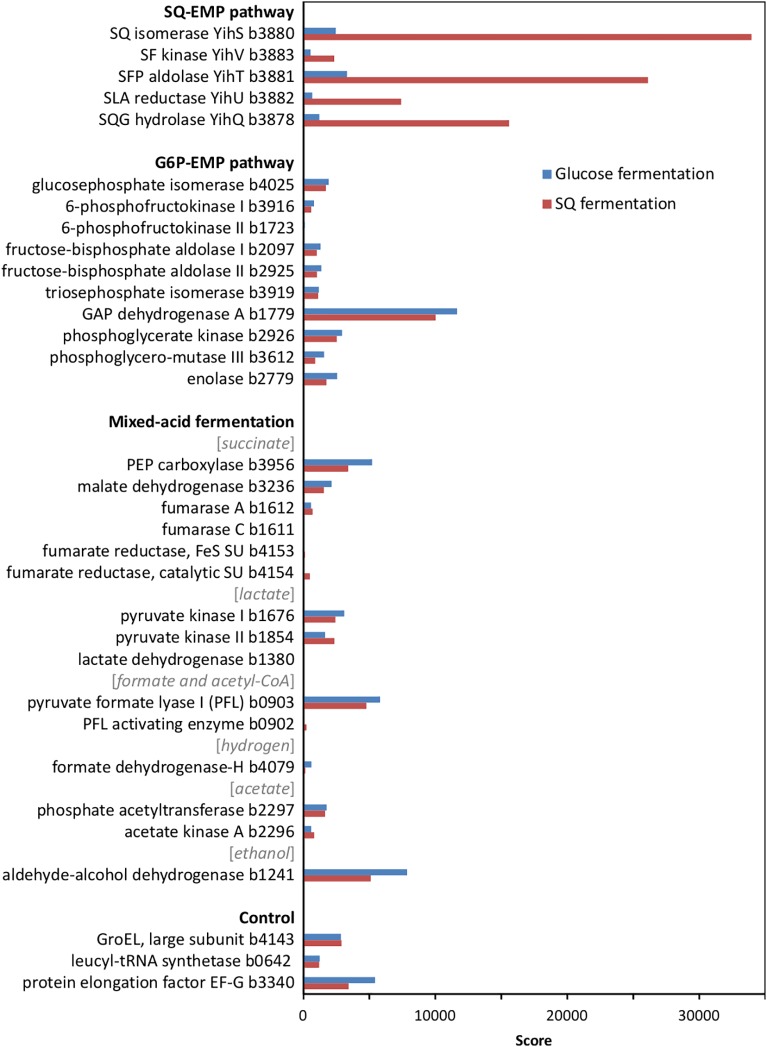
Total proteomic analysis comparing abundant soluble proteins in cell-free extracts of SQ- and glucose-fermenting *E. coli* cells. Shown are metabolic enzymes grouped according to the SQ Embden-Meyerhof-Parnas (SQ-EMP) pathway, the EMP pathway for glucose (G6P-EMP pathway) and to mixed-acid fermentation. Constitutively expressed proteins are shown for comparison (control). IMG locus tag numbers are given for each protein. Shown are data of a proteomic analysis replicated once. A higher score represents a more abundant protein.

At first, we confirmed the involvement of the SQ-EMP pathway in *E. coli* K-12 during fermentative growth with SQ. The knockout mutants used previously (Denger et al., 2014) with deleted SQ importer gene (Δ*yihO*), sulfosugar-metabolic genes (Δ*yihS*, Δ*yihV* and Δ*yihT*), or 3-sulfolactate reductase gene (Δ*yihU*), each failed to grow with SQ under fermentative conditions while these mutants grew by glucose fermentation (data not shown). In addition, differential proteomics confirmed proteins YihS, YihV, YihT, YihU and YihQ to be highly induced during SQ fermentation but not during glucose fermentation (Figure 2), as was observed previously for its aerobic growth with SQ (Denger et al., 2014).

Notably, no significant differences in the abundance of the EMP pathway enzymes for glucose (G6P-EMP pathway/gluconeogenesis) and of key enzymes involved in mixed-acid fermentation, such as for aldehyde-alcohol dehydrogenase, were detected by proteomics (Figure 2).

**FIGURE 3:**
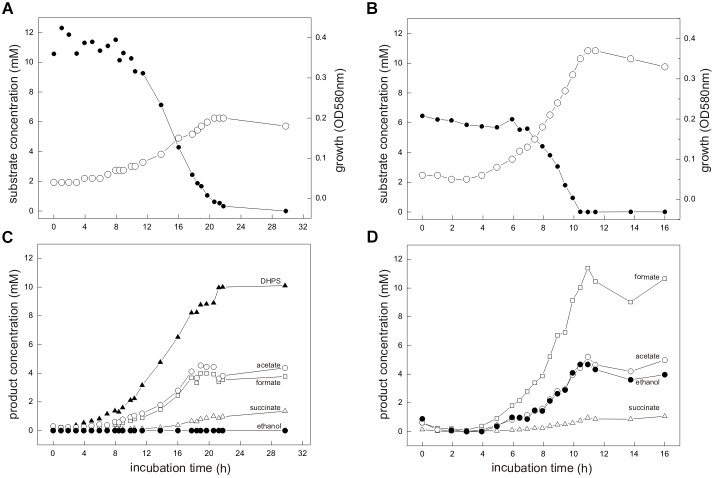
**(A–D)** *E. coli* K-12 growing in pure culture under fermentative conditions with SQ **(A,C)** and glucose **(B,D)**. For the growth experiment shown, 12 mM SQ or 6 mM glucose was used, so that both cultures had access to the same amount of C_3_-carbon (DHAP/GAP) for growth through the SQ-EMP and G6P-EMP pathways, respectively (i.e., 1 mol DHAP per mol of SQ, compared to 2 mol DHAP/GAP per mol of glucose; see Figure 1). **(A,B)** Biomass formation shown here as optical density (OD_580*nm*_), open circles; SQ or glucose disappearance, solid circles. **(C,D)** product formation (mM); formate, open square; acetate, open circle; ethanol, solid circle; succinate, open triangle; DHPS, solid triangle. For both cultures, lactate and H_2_ production was not detectable and therefore this data was omitted in this illustration. The cultures were incubated in 60 ml serum flasks with rubber stoppers containing, initially, 25 ml culture fluid and 35 ml N_2_/CO_2_ gas in the headspace. At each time interval, 1.0 ml of sample was removed with a syringe. The growth experiment was replicated once when sampled in such detail, and at least three times in smaller scale when evaluating only the outgrown cultures (t_*end*_) (see Figure 4 and Supplementary Table S1).

For the aerobic growth of *E. coli* with SQ (Denger et al., 2014), the molar growth yield is approximately 50% of that for glucose, which reflects that only half of the SQ-carbon can be made available for energy conservation (aerobic respiration) and biomass growth (assimilation) through the SQ-EMP pathway (via the DHAP/GAP), whereas the sulfonated C_3_-half is excreted (as DHPS). Therefore, we routinely supplied equivalent amounts of accessible substrate-carbon to our cultures for comparative growth experiments, i.e., 12 mM SQ and 6 mM glucose, respectively, for which a growth experiment sampled in great detail is shown in Figure 3.

**FIGURE 4:**
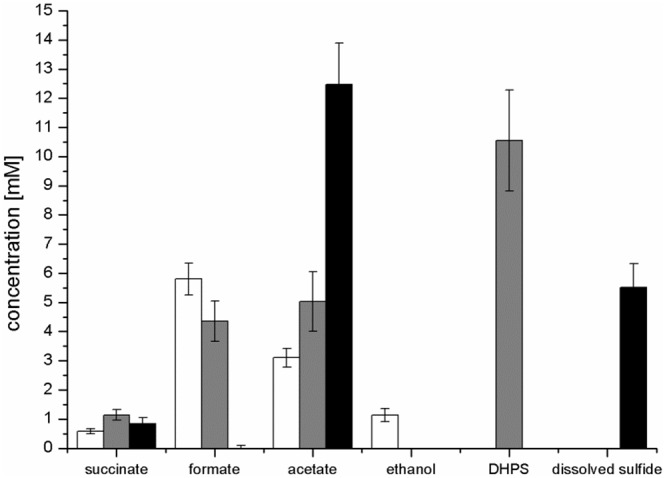
Concentration of products detected after growth of *E. coli* in pure culture with glucose (white bars) or SQ (gray bars) and after growth with SQ in co-culture with a DHPS-degrading *Desulfovibrio* sp. strain (black bars). The cultures were incubated in 25 ml culture tubes containing 10 ml culture fluid containing 12 mM SQ or 6 mM glucose and 15 ml N_2_/CO_2_ gas in the headspace; samples were collected when the cultures had entered stationary phase (t_*end*_). For a calculation of the carbon and electron recoveries for mixed-acid fermentation of SQ and glucose by *E. coli* in pure culture, see Supplementary Table S1. The data represents the mean of at least three growth experiments; the SD is indicated.

Strain K-12 exhibited a lower growth rate during SQ fermentation (μ = 0.1 h^-1^) in comparison to glucose fermentation (μ = 0.27 h^-1^) (Figures 3A,B); a lower growth rate with SQ compared to glucose was also observed for its aerobic growth (μ = 0.13 and 0.5 h^-1^, respectively (Denger et al., 2014)). Further, in comparison to aerobic respiration, only approximately 10–15% of total biomass (estimated as total protein content) was formed with SQ and glucose under fermentative conditions, which reflects that during fermentations the vast majority of the substrate-carbon is allocated to the production of electron acceptors. The molar growth yield for carbon determined for SQ fermentation in comparison to glucose fermentation, however, was consistently less than 50% (approximately 35–40% as estimated by total protein) and, thus, not equal when growth with equivalent amounts of accessible substrate-carbon was compared (12 mM SQ and 6 mM glucose), which is illustrated in Figures 3A,C by the different final optical densities observed. The replicate growth experiments in smaller scale (10 ml), which were sampled only after the growth (t_*end*_), confirmed this pattern, inclusive the fermentation products formed (see below) (Figure 4 and Supplementary Table S1); these results were independent of the addition of Ti(III)NTA as reducing agent.

Mixed-acid fermentation of glucose under our cultivation conditions yielded formate, acetate, ethanol and succinate as products (Figures 3D, 4 and Supplementary Table S1), as detected by HPLC. There was no lactate or any other fermentation product detectable (see Section “Materials and Methods”) in the culture fluid, and there was no H_2_ in the headspace in relevant amounts, as determined by GC (detection limit 4 μM). The SQ fermentation (Figures 3D, 4 and Supplementary Table S1) yielded quantitative amounts of DHPS, but no ethanol, more acetate and less formate as well as more succinate, when compared to glucose fermentation (cf. Figures 3C,D, 4), as illustrated also by the dissimilation equation calculated for SQ fermentation in comparison to glucose fermentation under our growth conditions (see Supplementary Table S1). There was no other fermentation product detectable in the culture fluid and no H_2_ in the headspace. From this data (Figure 4 and Supplementary Table S1), a carbon recovery of 82% and an electron recovery of 85% was calculated for SQ fermentation.

In conclusion, we demonstrated that *E. coli* K-12 is capable of utilizing SQ as sole substrate also for anaerobic growth through mixed-acid fermentation via the SQ-EMP pathway, for which the majority of the acquired C_3_-carbon is allocated to fermentation products, as in the case of mixed-acid fermentation of glucose. With the SQ-EMP pathway, however, *E. coli* employs the metabolite SLA as an additional electron acceptor for NAD^+^ regeneration, using the NADH-dependent reductase YihU (Figure 1A). This is reflected by the different ratio of fermentation products observed for SQ fermentation in comparison to glucose fermentation (Figures 3, 4): the different ratio of formate/succinate suggests that with SLA as additional electron acceptor, more of the phosphoenolpyruvate (PEP) is funneled to fumarate reduction for NAD^+^ regeneration (and proton pumping), rather than to acetyl-CoA and formate. The different ratio of acetate/ethanol, however, suggests that this acetyl-CoA is then fed only into the acetate-arm of the pathway for ATP conservation (see Figure 1A) while the ethanol-arm for NAD^+^ regeneration (aldehyde-alcohol dehydrogenase) is inactive. The observation that the molar growth yield for SQ fermentation is only approx. 35–40% of that for glucose is reflected by the carbon balance determined (see Supplementary Table S1), which suggests that a higher proportion of the accessible carbon was invested into dissimilation during growth with SQ in comparison to glucose (as indicated by the lower molar growth yield for dissimilated substrate, see Supplementary Table S1). While it appeared that SQ-fermentative growth is less energy yielding than initially expected, the exact reason remains unclear. It might point at an interesting tradeoff between metabolic yield (or metabolic rate) relative to the investment cost for enzyme production (protein synthesis) (e.g., Flamholz et al., 2013; Stettner and Segre, 2013), given the observed high expression of the SQ-EMP pathway enzymes in fermenting cells (see Figure 2) as well as in aerobically respiring cells (as seen previously on 2D-gels (Denger et al., 2014)). For example, a higher investment of the resources into protein synthesis for maintaining the SQ-EMP pathway flux relative to the G6P-EMP pathway flux (which yields twice the amount of DHAP/GAP per flux) might weight higher during fermentative substrate-level phosphorylation (with its low ATP yield) in comparison to oxidative phosphorylation under aerobic conditions (high ATP yield). Unfortunately, we are as yet unable to conduct kinetic experiments with the key SQ-EMP pathway enzymes in comparison to the G6P-EMP pathway enzymes, because the appropriate substrates (SF, SFP, SLA) are not available.

### Enrichments and Isolation of an SQ-Fermenting *Citrobacter* sp. Strain and a DHPS-Fermenting *Desulfovibrio* sp. Strain

In order to initially examine the occurrence of anaerobic SQ- and DHPS-degrading bacteria and when aiming at isolating new SQ- and/or DHPS-metabolizing strains, we set up enrichment cultures with inocula from different anaerobic environmental habitats: from a sewage treatment plant, a biogas plant, garden compost and Lake Constance sediment. All enrichment cultures, and the subcultures thereof, grew and degraded SQ completely and in some cultures transient DHPS formation was detected by HPLC (not shown). Sulfide production, as detected by its smell, was confirmed for all enrichments. The fastest growing enrichments (sewage sludge and compost) were purified via agar dilution series using SQ as substrate and colony picking. In these agar shakes, we observed two colony types: light beige colonies and dark beige colonies. One representative light beige colony (enriched from compost) was purified further using SQ-containing agar dilution and colony picking into SQ-containing liquid medium. The isolate degraded SQ completely and produced DHPS. It was identified by 16S rRNA gene sequencing as a *Citrobacter* sp. strain (99% identity with *Citrobacter freundii*). Thus, it represented another anaerobic SQ-degrading, DHPS-producing enterobacterium, such as *E. coli* K-12. In the genomes of other *Citrobacter* species, the SQ-EMP pathway is encoded (as examined via the JGI-IMG platform). Hence, because the isolate most likely employed also the SQ-EMP pathway, it was not further examined.

The other, dark beige colonies, however, never grew when transferred into SQ-containing liquid medium, but grew in DHPS-containing liquid medium. The DHPS was completely degraded and sulfide was formed, even when sulfate as additional electron acceptor had been omitted in these cultures (see below). One representative colony enriched from sewage sludge was purified further via DHPS-containing agar dilution and colony picking into DHPS-containing liquid medium without additional sulfate. The isolate was identified by 16S rRNA gene sequencing as a *Desulfovibrio* sp. strain (99% identity with *D. alcoholivorans*).

**FIGURE 5:**
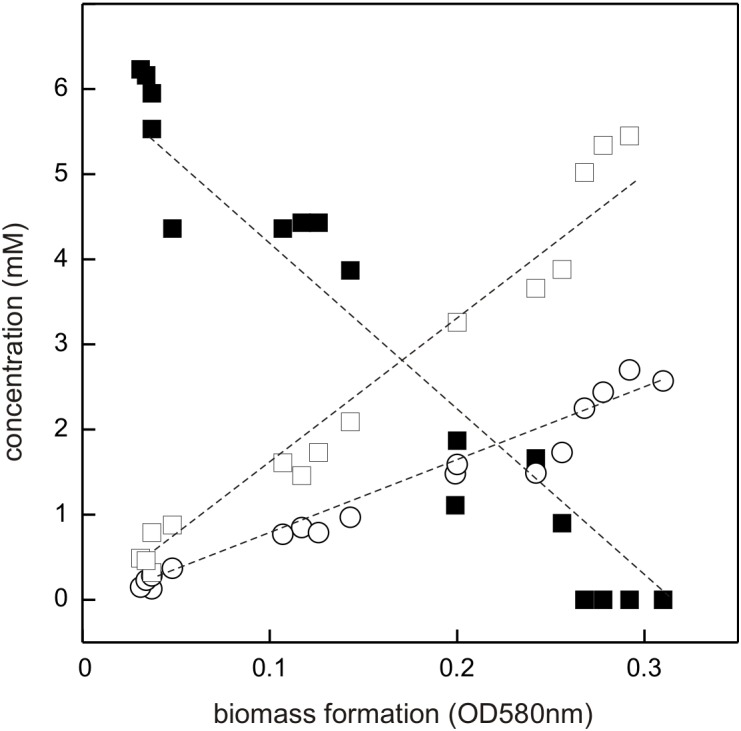
Linearized growth plot illustrating substrate disappearance and formation of acetate and sulfide during growth of *Desulfovibrio* sp. strain DF1 with DHPS. Concentrations of DHPS (solid square), acetate (open square) and of dissolved sulfide/bisulfide in the culture fluid (open circle) were determined and the values plotted against biomass formation (OD 580 nm). Note that we did not determine the fraction of H_2_S dissolved in the gas phase of the culture vessel, which is thus missing in the overall sulfur quantitation. No sulfate or sulfite were detectable in the culture at any time during growth (not shown). This growth experiment was conducted once when sampled in such detail; the substrate disappearance, biomass and product formation were confirmed in smaller scale when evaluating only the outgrown cultures (t_*end*_) (see Figure 4).

The DHPS-degrading *Desulfovibrio* sp. isolate was termed strain DF1 (“DHPS-fermenting isolate no. 1”), was draft-genome sequenced and its growth with DHPS as sole substrate in the absence of sulfate as electron acceptor was characterized in detail. As illustrated by a linearized growth plot in Figure 5, the DHPS disappeared concomitant with biomass formation and nearly quantitative amounts of acetate were formed.

Further, there was sulfide produced during growth, as detected by the dissolved sulfide/bisulfide in samples of culture fluid, though not in quantitative amounts relative to substrate disappearance and acetate formation (Figure 5). This is because of the partitioning of the sulfide between the liquid phase of the culture and its gas phase (as H_2_S gas, some of which is also lost during the sampling). Notably, the nearly quantitative conversion of DHPS to acetate, and the absence of any other product in the culture fluid (as determined by Aminex HPLC; see Section “Materials and Methods”), implied that no other (sulfonated) degradation product of DHPS was formed. The growth rate of *Desulfovibrio* sp. strain DF1 with DHPS was approx. μ = 0.01 h^-1^.

In conclusion, *Desulfovibrio* sp. strain DF1 produced sulfide during DHPS utilization in the absence of added sulfate as electron acceptor. This implied that strain DF1 utilizes the carbon-moiety of DHPS as a source of carbon and electrons and its sulfonate-moiety as electron acceptor, hence, by a metabolism in analogy to the described respiration with taurine (2-aminoethanesulfonate) by *Bilophila wadsworthia* (Laue et al., 1997) or isethionate (2-hydroxyethanesulfonate) by *Desulfovibrio* spp. (Lie et al., 1996): the sulfite released by a desulfonation of an organosulfonate substrate, such as taurine, isethionate or DHPS, is utilized as electron acceptor in respiration and reduced to sulfide by dissimilatory sulfite reductase (Dsr), as illustrated in Figure 1B for DHPS. Compared to sulfate-reducing bacteria (Muyzer and Stams, 2008), such “organosulfonate respiration” confers the ability to occupy distinct metabolic niches and there is no expenditure of ATP necessary for generating sulfite from sulfate as electron acceptor (see Figure 1B).

For *Desulfovibrio* sp. strain DF1 and DHPS as substrate (Figure 1B), the respiratory energy metabolism is represented by the following equation,

$$C_{3}H_{7}O_{5}S^{-}\to C_{2}H_{3}O_{2}^{-}+{\mathrm{HCO}}_{3}^{-}+{\mathrm{HS}}^{-}+2H^{+}$$

which through the following redox half-reactions represents a fermentation of DHPS,

Oxidation of DHPS−carbon: −2→+4+6[H]

Reduction of DHPS−sulfur: +4+6[H]→−2.

### Conversion of SQ via DHPS to Sulfide by a Co-culture of *E. coli* K-12 and *Desulfovibrio* sp. Strain DF1

**FIGURE 6:**
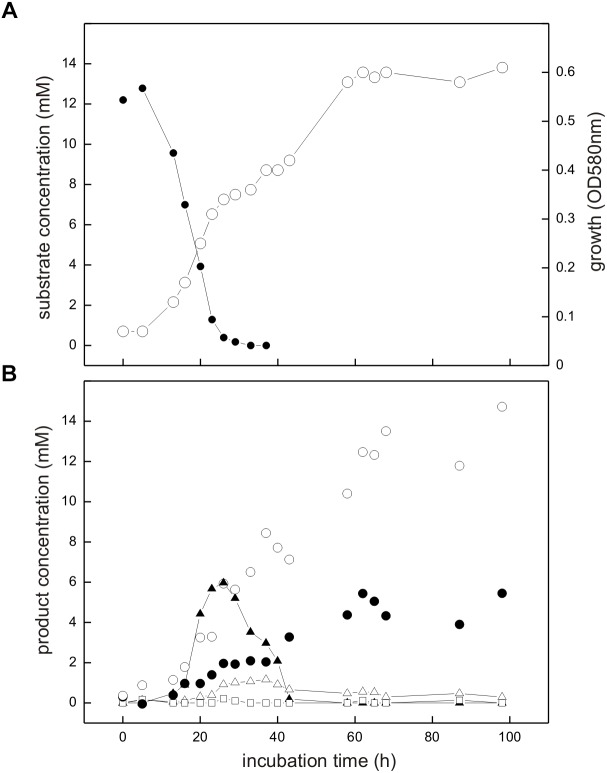
Co-culture of *E. coli* K-12 and *Desulfovibrio* sp. strain DF1 growing with SQ under fermentative conditions. The culture was incubated in a 60 ml serum flask containing, initially, 25 ml culture fluid and 35 ml N_2_/CO_2_ gas in the headspace; at each time interval, 1.0 ml of sample was removed with a syringe. **(A)** Biomass formation as monitored by optical density (OD_580*nm*_), open circles; disappearance of SQ, solid circles. **(B)** Degradation products as detected in the culture supernatant; DHPS, solid triangles; acetate, open circles; dissolved sulfide, solid circles; succinate, open triangles; formate, open squares. The growth experiment was replicated once when sampled in such detail; substrate disappearance, biomass and product formation was confirmed in smaller scale when evaluating only the outgrown co-cultures (t_*end*_) (see Figure 4).

The co-culture consisting of *E. coli* K-12 and *Desulfovibrio* sp. strain DF1 growing with SQ released reduced inorganic sulfur, i.e., sulfide, as shown in Figure 6.

Strains K-12 and DF1 were co-inoculated into culture medium containing 12 mM SQ and the growth of the culture was monitored. A two-phasic growth was observed, as illustrated by the overall optical-density readings obtained (Figure 6A). After the first growth phase (after approx. 25 h incubation time), SQ (12.2 mM) had disappeared completely (Figure 6A) and DHPS was formed, though not in quantitative amounts (approx. 6 mM DHPS) (Figure 6B). Furthermore, acetate and succinate were produced but no formate was detectable (Figure 6B) in comparison to growth of *E. coli* with SQ in pure culture (see Figures 3, 4). In addition, sulfide formation (up to approx. 2 mM) was observed already in the first growth phase (Figure 6B). After the following growth phase (after approx. 60 h incubation time; Figure 6A), DHPS had disappeared completely from the culture fluid and additional sulfide and acetate were formed; some of the succinate had also disappeared (Figure 6B; see also Figure 4). Overall, these observations reflect the growth of SQ-degrading, DHPS-excreting *E. coli* predominantly in the first growth phase and of DHPS-utilizing, sulfide-producing *Desulfovibrio* sp. strain DF1 predominantly in the second growth phase. The absence of formate in the co-culture, as produced by *E. coli* during SQ-fermentation in pure culture (see Figure 4), can be explained by its utilization as an additional electron donor by *Desulfovibrio* sp. strain DF1.

### Identification of the DHPS Desulfonation Pathway in *Desulfovibrio* sp. Strain DF1

We next studied the DHPS-desulfonation pathway in *Desulfovibrio* sp. strain DF1. Firstly, cell-free extracts of *Desulfovibrio* sp. strain DF1 grown with DHPS in comparison to lactate/sulfate as electron donor/acceptor were examined in respect to detectable enzyme activities. With DHPS as substrate, a high NAD^+^-dependent dehydrogenase activity (up to 225 mU per mg protein at its pH optimum, pH 10.5) was detected in the soluble protein fraction. The product of the reaction was identified as SLA by HPLC-MS. No activity was observed in extracts of lactate/sulfate-grown cells, thus, the DHPS dehydrogenase was specifically and highly induced during growth with DHPS. Furthermore, sulfite was formed after addition of SL as substrate to the soluble protein fraction of DHPS-grown cells (up to 38 mU/mg), but not after addition of DHPS. There was no sulfite formation after addition of SL to cell-free extracts of lactate/sulfate-grown cells. Hence, a DHPS dehydrogenase and a SL-desulfonating enzyme were found to be specifically induced during growth with DHPS in *Desulfovibrio* sp. strain DF1.

**FIGURE 7:**
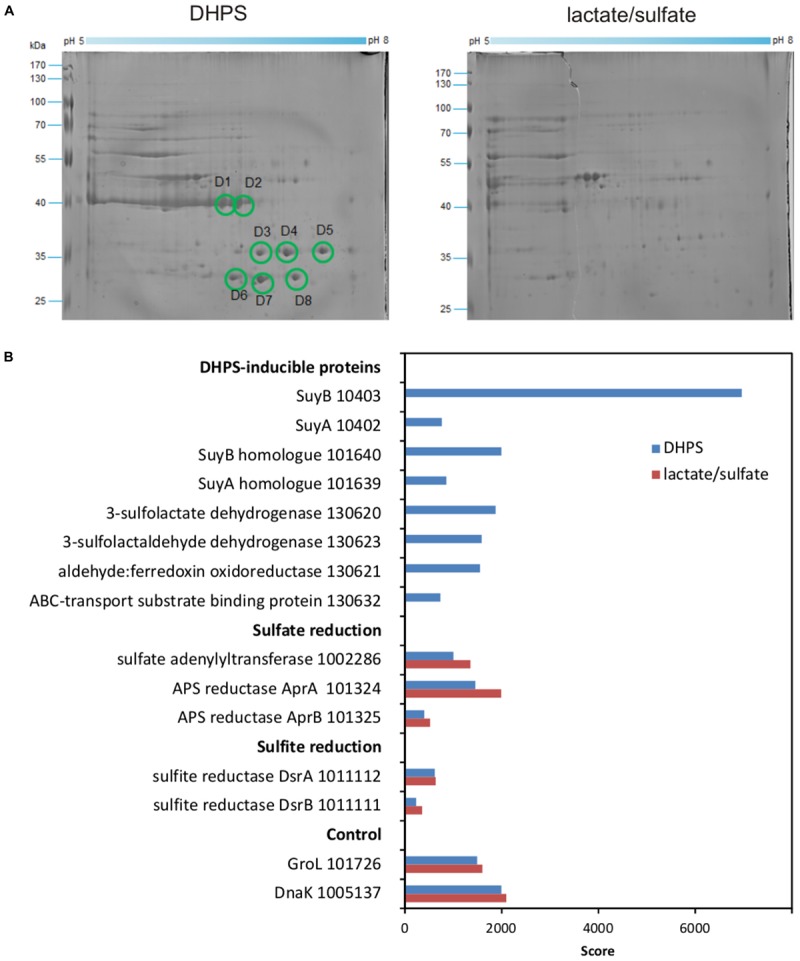
**(A,B)** Proteomic identification of DHPS-inducible proteins in cell-free extracts of DHPS-grown *Desulfovibrio* sp. strain DF1 in comparison to lactate/sulfate-grown cells. **(A)** Two-dimensional PAGE gels. All prominent protein spots (labeled D1–D8) that were found exclusively on the gel for DHPS-grown cells were excised and identified by peptide fingerprinting mass spectrometry. Their identities are described in the main text. The identification of the protein spots was done once. **(B)** Total-proteomics results. Shown are proteins which were highly abundant exclusively in DHPS-grown cell extracts in comparison to constitutively expressed proteins for sulfate reduction, sulfite reduction and other cellular functions (control). IMG locus tag numbers are given for each protein. The results were replicated once when starting from an independent growth experiment.

We then proceeded with differential proteomics in order to identify these inducible enzymes (Denger et al., 2014; Felux et al., 2015). An annotated draft-genome sequence of strain DF1 was generated for proteomics using Illumina HiSeq sequencing and JGI’s IMG annotation pipeline (Felux et al., 2015); the annotation is publicly available under IMG Project ID Gp0153975. 2D-gel-based proteomics (Figure 7A) as well as total proteomics (Figure 7B) were performed for strain DF1 cells grown with DHPS in comparison to cells grown with lactate/sulfate.

For the 2D-gels (Figure 7A), major protein spots visible only for DHPS-grown cells indicated abundant proteins specifically induced during growth with DHPS. These were identified by peptide-fingerprinting mass spectrometry.

The most prominent spots on the 2D-gel for DHPS-grown cells, spots D1 and D2 (Figure 7A), exhibited about the same molecular weight but were resolved at different isoelectric points. The two protein spots identified a gene (IMG locus tag Ga0134130_10403) (in the following, the IMG locus tag prefix Ga0134130_ is omitted) annotated to encode a large, catalytic subunit (SuyB) of a desulfonating enzyme, 3-sulfolactate sulfite-lyase (SuyAB). Hence, protein 10403 was the prime candidate for the inducible SL-desulfonating enzyme observed in cell-free extracts of *Desulfovibrio* sp. strain DF1 (see above). The total proteomics data (Figure 7B) confirmed and expanded on these results. The prominent SuyB-component 10403 of the sulfite-lyase was detected as the highest scoring protein specifically in DHPS-grown cells, but not in lactate/sulfate-grown cells, as well as an homologous *suyB* gene (87.6% identity) encoded elsewhere in the genome (with lower score, IMG locus tag number 101640; see Figure 7B). In addition, the SuyA component gene 10402 encoded directly upstream of the SuyB gene was also identified (and its paralog 101639; 73.4% identity) specifically in DHPS-grown cells (Figure 7B).

Two other prominent spots, D7 and D8 (Figure 7A) observed at about the same molecular weight but different isoelectric points, were identified as protein 130620, annotated as NAD^+^-dependent *beta*-hydroxyacid/3-hydroxyisobutyrate dehydrogenase: it belongs to the same group of orthologs (Clusters of Orthologous Group COG2084) as the characterized oxidoreductase catalyzing the reverse reaction in *E. coli* K-12, that is, SLA reductase YihU; their overall sequence identity, however, is low (27%). Thus, protein 130620 (termed DhpA) was a prime candidate for the enzyme converting DHPS to SLA in strain DF1. DhpA was confirmed to be a highly abundant, DHPS-inducible protein by total proteomics (Figure 7B).

Spots D3–D6 identified gene 130632, coding for a substrate-binding protein of a predicted organosulfonate ABC-type transport system (SsuA/TauT family), thus, a candidate for DHPS transport. It is encoded together with each two candidate genes for ATP-binding and permease components on the same contig as the candidate DHPS dehydrogenase (DhpA) (Figure 1C). This soluble substrate-binding protein was also detected in DHPS-grown cells by total proteomics (Figure 7B).

Another protein, 130623, annotated as succinate semialdehyde dehydrogenase and co-encoded with the DhpA gene, appeared to be highly abundant in DHPS-grown cells by total proteomics (Figure 7B). This protein belongs to the same COG and is 61% identical to the characterized NAD^+^-dependent SLA dehydrogenase of the SQ-pathway of *P. putida* SQ1 (locus tag PpSQ1_00088) (Felux et al., 2015). Hence, protein 130623 encodes for the prime candidate for a dehydrogenase converting SLA to SL (and was termed SlaB; Figure 1B). In addition, another DHPS-inducible protein co-encoded with the genes for DhpA and SlaB was detected by total proteomics (protein 130621; Figure 7B), predicted as aldehyde:ferredoxin oxidoreductase (COG2414) (gene labeled as oxidored. in Figure 1C); it may represent a second candidate for an oxidation of SLA to SL.

In conclusion, the strongly DHPS-inducible proteins identified by proteomics and their annotation in accordance to the activities of DHPS-inducible enzymes detected in cell-free extracts, suggested a desulfonation pathway for DHPS in *Desulfovibrio* sp. strain DF1 (see Figure 1B) in analogy to the pathway described for the aerobic *C. pinatubonensis* JMP134 (Mayer et al., 2010): DHPS is transported into the cells and oxidized in two steps via SLA to SL by two NAD^+^-dependent dehydrogenases in strain DF1 (DhpA and SlaB; proteins 130620 and 130623, respectively); the oxidation of SLA may involve also a ferredoxin-coupled aldehyde dehydrogenase (protein 130621). Then, SL is desulfonated by SuyAB (proteins 10402 and 10403) yielding pyruvate as a carbon and energy source and sulfite as the terminal electron acceptor.

### Reconstruction of a DHPS-Desulfonation Pathway by Three Recombinant Enzymes Overexpressed in *E. coli*

**FIGURE 8:**
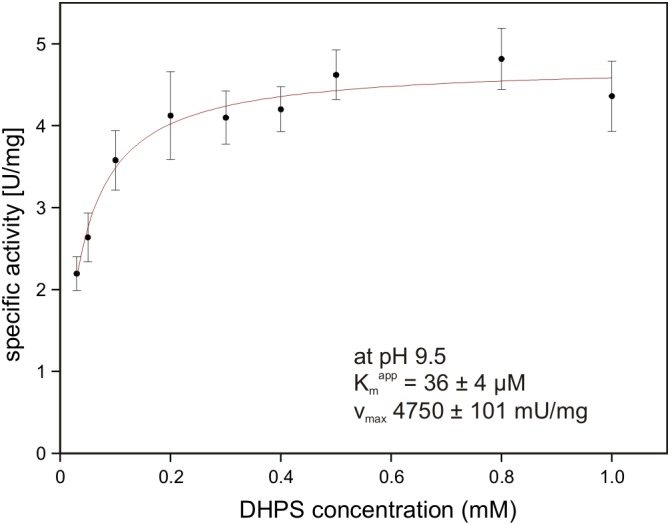
NAD^+^-dependent DHPS oxidation by recombinant dehydrogenase DhpA. The reactions contained 0.85 μg/ml DhpA in 50 mM Tris–HCl buffer at pH 9.5. The DHPS concentration was varied in the presence of 10 mM NAD^+^ and the initial rates of NADH formation were determined spectrophotometrically as increase of absorbance at 340 nm. The data shown represents the mean ± SD of three technical replicates.

The gene for the candidate DHPS dehydrogenase DhpA (annotated as 3-hydroxyisobutyrate dehydrogenase) was cloned and heterologously overexpressed, and the protein was purified by His-Tag affinity chromatography. The purified protein produced a single band on a SDS gel at around 33 kDa (calculated mass: 33.3 kDa including the His-tag) (Supplementary Figure S1). No activity was observed with 3-hydroxyisobutyrate as substrate, but the enzyme oxidized DHPS with NAD^+^ but not with NADP^+^ as cosubstrate. The product of the reaction was confirmed as SLA by HPLC-MS using SLA as standard that had been generated by SLA reductase YihU of *E. coli* catalyzing the reverse reaction with DHPS (Denger et al., 2014). The $K_{m}^{\mathrm{app}}$ for DHPS was observed at 36 ± 4 μM (*V*_*max*_ 4.75 ± 0.10 U/mg protein) at pH 9.5 (Figure 8) and at 1.54 ± 0.33 mM (*V*_*max*_ 3.15 ± 0.38 U/mg) at pH 7.5. Hence, we demonstrated that DhpA is a NAD^+^-dependent SLA-producing DHPS dehydrogenase.

**FIGURE 9:**
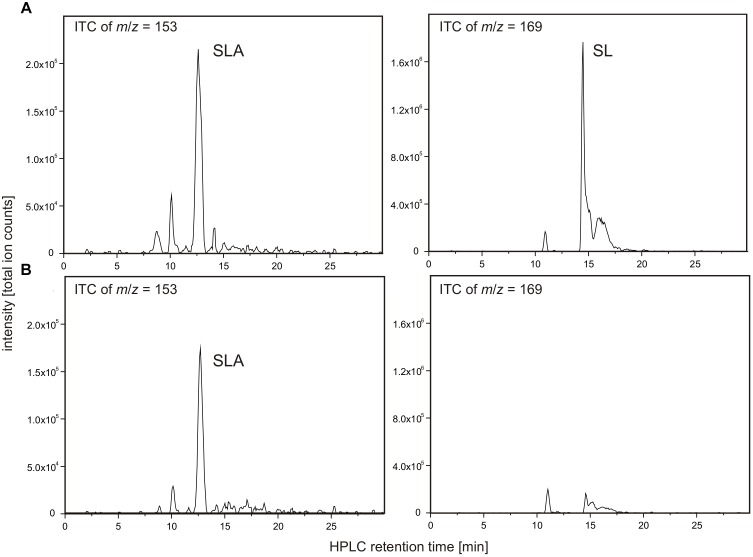
NAD^+^-dependent SLA oxidation to SL by recombinant dehydrogenase SlaB. Substrate SLA for candidate SLA dehydrogenase SlaB was generated from DHPS by coupling of the DhpA reaction. The reactions contained 5 μg/ml DhpA, 16.5 μg/ml SlaB, 5 mM DHPS and 10 mM NAD^+^ in 50 mM Tris–HCl buffer at pH 9.0. Shown are HPLC-MS ion trace chromatograms (ITC) of samples taken after the reactions when screening for the quasimolecular ions of SLA (left panel) and SL (right panel). **(A)** Ion trace chromatograms demonstrating formation of SLA and SL in a reaction containing both enzymes, SlaB and DhpA. **(B)** Ion trace chromatograms of control reaction containing active DhpA and heat-inactivated SlaB. The results **(A,B)** were replicated once with independently prepared enzyme preparations. No quantitative measurements were possible for SLA due to the lack of an analytical standard.

Recombinant candidate SLA dehydrogenase SlaB (annotated as succinate semialdehyde dehydrogenase) on an SDS gel produced a single band as expected at around 55 kDa (calculated mass: 54.9 kDa including the His-tag) (Supplementary Figure S1). Because the substrate SLA is not commercially available, the candidate SLA dehydrogenase was tested in reactions coupled with SLA-producing DhpA and DHPS as substrate (Figure 9). Hence, we could not determine the kinetic parameters of the SLA dehydrogenase.

As illustrated in Figure 9A, the combination of DhpA and SlaB produced SL from DHPS with NAD^+^ as co-substrate, compared to, for example, an assay for which heat-inactivated SlaB of *Desulfovibrio* was used. Hence, we confirmed that SlaB is a NAD^+^-dependent, SL-producing SLA dehydrogenase. Furthermore, we confirmed that the NAD^+^-dependent DHPS dehydrogenase DhpA produces SLA but does not catalyze its oxidation to SL. The identified aldehyde:ferredoxin oxidoreductase (locus tag Ga0134130_130621) was also produced recombinantly, purified, and tested as candidate SLA dehydrogenase under anoxic conditions with benzylviologen (replacing ferredoxin) as the electron acceptor and with SLA as substrate generated from DHPS by dehydrogenase DhpA in the presence of NAD^+^. We could not detect benzylviologen-reducing activity in the spectrophotometrical assays when the SLA was formed through DhpA, and we detected no SL formation by HPLC, under the conditions we used. The enzyme showed also no activity with lactaldehyde or acetaldehyde as substrates. Hence, the role of this inducible protein in *Desulfovibrio* sp. strain DF1 remained unclear.

**FIGURE 10:**
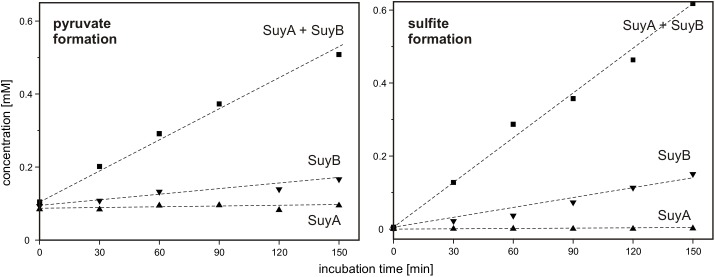
Cleavage of SL into pyruvate and sulfite by recombinant SuyAB proteins. The reactions contained 150 μg/ml SuyA and/or 600 μg/ml SuyB and 2.5 mM SL in 100 mM anoxic MOPS buffer at pH 7.0, containing 10% glycerol and 1 mM FeCl_2_. Formation of pyruvate and sulfite was detected by HPLC-UV/Vis in samples taken discontinuously and after derivatization with DNPH and NAM, respectively (see Section “Materials and Methods”). Left panel: pyruvate formation. Right panel: sulfite formation. Upward triangles, reaction containing SuyA; downward triangles, reaction containing SuyB; squares, reactions containing both subunits, SuyA and B. The results were replicated once with an independently prepared enzyme preparation.

Finally, SuyA and SuyB were separately expressed and purified. The proteins on an SDS gel produced bands as expected at around 15 and 46 kDa (calculated mass: 14.5 and 45.9 kDa including the His-tags), respectively (Supplementary Figure S1). Desulfonation reactions with SL as substrate were followed by formation of sulfite and pyruvate, as determined each by HPLC in samples that were taken at intervals from the reactions, derivatized and analyzed (Figure 10): the cleavage of SL to sulfite and pyruvate was detected after addition of both subunits, SuyA and B, and a lower but significant activity after addition of the SuyB subunit only; no activity was detectable when only the SuyA subunit was added.

Hence, we confirmed that SuyAB is a SL sulfite-lyase, with SuyB being catalytically active. For reactions with the holoenzyme (SuyAB) a specific activity of 4.5 mU/mg was determined, and for SuyB alone, a specific activity of 1.9 mU/mg.

### Phylogenetic Distribution of SuyB Among Sulfate/Sulfite-Reducing Bacteria

**FIGURE 11:**
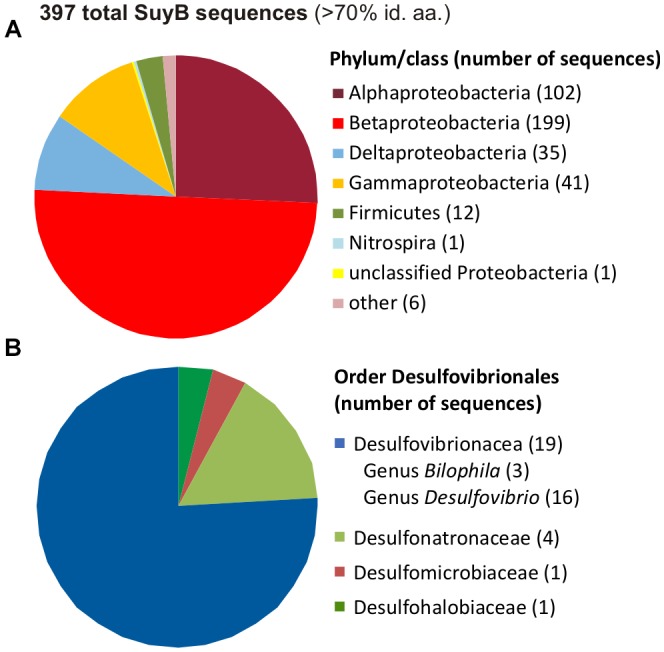
Distribution of SuyB in bacterial genomes. Pie charts illustrating the distribution of 397 BLAST hits retrieved from the non-redundant database when searching for SuyB homologs with a threshold of >70% on the phylum/class level **(A)** and in the order Desulfovibrionales **(B)**.

A search for putative genes for SuyB in microbial genomes retrieved 397 hits in the NCBI non-redundant protein database with each >70% amino acid identity to the *Desulfovibrio* sp. strain DF1 sequence (Figure 11).

Most of these sequences were found in genomes of betaproteobacteria (199 genomes), alphaproteobacteria (102 genomes) and gammaproteobacteria (41 genomes), hence, in organisms that most likely employ SuyAB in aerobic and facultatively anaerobic catabolism of DHPS and/or cysteate or SL (see Section “Introduction”) as carbon and energy source(s), while the sulfite may be oxidized to sulfate, such as in aerobic *C. pinatubonensis* JMP134 (Mayer et al., 2010) and *Paracoccus pantotrophus* NKNCYSA (Rein et al., 2005).

Furthermore, 35 genomes of deltaproteobacteria and 12 genomes of Firmicutes contained *suyB* (as well as *suyA*) genes. For the largest group of sulfate/sulfite-reducing bacteria among the deltaproteobacteria, *suyB* (and *suyA*) genes were found in the sulfate/sulfite-reducing bacteria of the human gut microbiota and HMP reference strains *Desulfovibrio* sp. 3.1.syn3 and 6.1.46AFFAA, in *D. fairfieldensis* CCUG45958, and in all three available *B. wadsworthia* strains. Furthermore, candidate genes were found, for example, in *Desulfovibrio desulfuricans* DSM 642, *Desulfovibrio litoralis* DSM 11393, *Desulfonatronum lacustre* DSM 10312, *Desulfomicrobium baculatum* DSM 4028, *Desulfobulbus oralis* 041 and *Desulfomonile tiedjei* DSM 6799. For the second largest group of sulfate-reducing bacteria among the Firmicutes, candidate genes were found, for example, in *Desulfotomaculum ruminis* DSM 2154, *Desulfotomaculum arcticum* DSM 17038, *Desulfitobacterium hafniense* DSM 12704 and *Desulfosporosinus acidiphilus* DSM 22704. Hence, these sulfate/sulfite-reducing bacterial strains likely employ SuyAB also for a desulfonation of SL and organosulfonate respiration, when utilizing DHPS, SL and/or cysteate or yet unknown organosulfonates that may be metabolized via SL and SuyAB. Notably, our preliminary tests with *B. wadsworthia* (unpublished results) revealed its ability to utilize SL as electron acceptor with lactate as electron donor, and our preliminary proteomic analysis confirmed a strong induction of SuyAB.

## General Conclusion

Aerobic bacterial degradation of SQ has been reported almost 60 years ago (Benson and Shibuya, 1961) and the formation of DHPS or SL during its primary degradation 15 years ago (Roy et al., 2003). The latter observation implied an involvement of two-tier bacterial consortia for closing the sulfur cycle for SQ, as has been demonstrated in 2012 (Denger et al., 2012). Further, the pathways for degradation of DHPS and/or SL to sulfate (e.g., Denger and Cook, 2010; Mayer et al., 2010) as well as for primary degradation of SQ to DHPS or SL (Denger et al., 2014; Felux et al., 2015) have been demonstrated each for aerobically respiring model bacteria. Our present knowledge on degradation of SQ, DHPS and SL has been recapitulated in a recent review (Goddard-Borger and Williams, 2017), however, the anaerobic bacterial degradation of SQ, potentially producing harmful H_2_S instead of sulfate, has never been addressed. Using a laboratory model community, we demonstrated for the first time bacteria and pathways for anaerobic bacterial SQ degradation concomitant with H_2_S production, which represents another novel, important link in the biogeochemical sulfur cycle.

We demonstrated also two novel types of energy metabolism in anaerobic bacteria. Firstly, *E. coli* K-12 catalyzes the fermentation of SQ to DHPS, succinate, acetate and formate, thus, a novel type of mixed-acid fermentation, for which the SQ-EMP pathway is employed (Figure 1A). The option to utilize SQ as carbon and energy (electron) source for both mixed-acid fermentation (e.g., Figures 3A,D) and aerobic respiration (Denger et al., 2014) is another example of the adaptations of *E. coli* to its commensal lifestyle in the intestinal tract (e.g., Conway and Cohen, 2015) as well as for its survival in extra-intestinal environments (e.g., van Elsas et al., 2011). Secondly, *Desulfovibrio* sp. strain DF1 catalyzes a fermentation of DHPS to acetate and sulfide which involves energy conservation through sulfite respiration: the sulfite generated by the DHPS desulfonation pathway is utilized as terminal sink for the electrons derived from the oxidation of the DHPS-carbon (Figure 1B). Hence, DHPS was identified as a fourth organic sulfite-donor for anaerobic respiration, in addition to taurine (Laue et al., 1997), isethionate and cysteate (Lie et al., 1996). We believe that sulfidogenic organosulfonate respiration is more widespread and abundant in microbiomes than initially was realized. For example, it might be employed also by archaea (Lin et al., 2015). The organosulfonate substrates are at least abundant in the environment, for example, in soils and in sediments (Autry and Fitzgerald, 1990; Vairavamurthy et al., 1994) and in gut microbiomes (see below). Clearly, a microbiomic examination of organosulfonate respiring microorganisms is needed to fully reveal their diversity, ecological niches, and significance in the sulfur cycle, for which the description of sulfur-metabolizing biochemical pathways and their enzymes and genes in laboratory model bacteria, as achieved in this study, is an essential prerequisite (Wasmund et al., 2017).

Sulfoquinovose is a relevant constituent of the vegetable diet of herbivores and omnivores, and sulfide production in the intestinal microbiome has many recognized and potential contributions to human health and disease (e.g., Carbonero et al., 2012; Singh and Lin, 2015): sulfide is a potent genotoxin and may contribute to the onset of colorectal cancer (Attene-Ramos et al., 2010); it can reduce disulfide bonds in the mucus layer of the gut epithelium, disrupting its barrier function and potentially playing a role in inflammatory bowel disease (IBD) (Ijssennagger et al., 2016); and it can induce antibiotic resistance, so that sulfide production by the gut microbiota may trigger blooms of opportunistic bacteria during antibiotic treatment (Shatalin et al., 2011). The occurrence of the genes for a DHPS/SL desulfonation pathway via SuyAB also in typical anaerobic sulfate/sulfite-reducing bacteria of the human gut microbiota may highlight an important role for respiration with DHPS and/or SL-sulfite also in this microbial habitat, e.g., in dependence on the dietary conditions of the host: a sulfidogenic metabolism driven by substrates derived of green-vegetable diet (SQ, DHPS, SL) would contrast the already recognized sulfidogenic taurine metabolism (e.g., by the opportunistic pathogen *B. wadsworthia*) in dependence on the consumption of a high-fat diet (high taurocholate) (Devkota et al., 2012) and/or high-meat (high taurine) diet (David et al., 2014). Notably, most recently microbial SL desulfonation has been associated with altered sulfur metabolism in the microbiome of pediatric IBD patients (Zhang et al., 2018). Hence, it is important to examine in future also in the human intestinal microbiome the pathways and bacterial consortia involved in sulfidogenic SQ degradation, in relation to H_2_S production, dietary conditions and human health. This will not only enable efforts to understand the biological roles of this metabolic activity in the human body, but may also open the door to new approaches to control intestinal H_2_S production.

## Author Contributions

AB, KD, and DSch conceived and designed the experiments. AB and KD performed the experiments. TH contributed the reagents. AB, KD, PF, NM, DSp, and DSch analyzed the data. AB, KD, and DSch wrote the manuscript. All authors approved the manuscript.

## Conflict of Interest Statement

The authors declare that the research was conducted in the absence of any commercial or financial relationships that could be construed as a potential conflict of interest.

## Footnotes

1 https://www.dsmz.de

2 https://img.jgi.doe.gov

3 https://jgi.doe.gov/user-program-info/pmo-overview/protocols-sample-preparation-information/

4 http://img.jgi.doe.gov/

5 www.proteomics-facility.uni-konstanz.de
