# Supplementary material for: Anaerobic Degradation of the Plant Sugar Sulfoquinovose Concomitant With H2S Production: Escherichia coli K-12 and Desulfovibrio sp. Strain DF1 as Co-culture Model
Source: Front Microbiol. 2018 Nov 27;9:2792. doi: 10.3389/fmicb.2018.02792 (PMC6278857; doi:10.3389/fmicb.2018.02792)
Supplement: Supplementary file 1 [file Table_1.pdf]

SUPPLEMENTARY MATERIAL

Frontiers in Microbiology 2018 | doi: 10.3389/fmicb.2018.02792

Anaerobic degradation of the plant sugar sulfoquinovose concomitant with H_2S production: *Escherichia coli* K-12 and *Desulfovibrio sp.* strain DF1 as co-culture model

Anna Burrichter[1,2], Karin Denger[1], Paolo Franchini[1], Thomas Huhn[2,3], Nicolai Müller[1], Dieter Spiteller[1,2] and David Schleheck[1,2*]

Affiliations:

[1] Department of Biology, University of Konstanz, Konstanz, Germany

[2] Konstanz Research School Chemical Biology, University of Konstanz, Konstanz, Germany

[3] Department of Chemistry, University of Konstanz, Konstanz, Germany

***Correspondence:** David Schleheck
Department of Biology and Konstanz Research School
Chemical Biology, University of Konstanz, D-78457 Konstanz
Tel.: +49-7531-88-4247
Email: david.schleheck@uni-konstanz.de

Table S1. Stoichiometry of fermentation of glucose and of SQ by *E. coli* K-12

	**Fermentative growth in 10 ml 6 mM glucose medium**	**Fermentative growth in 10 ml 12 mM SQ medium**
**Total protein** [mg]	0.724	0.556
**Total cell dry mass** [mg]	1.45	1.11
**Assimilation equation**	$17\ C_6H_{12}O_6 \rightarrow$ $24 <C_4H_7O_3> + 6\ CO_2 + 18\ H_2O$	$17\ C_6H_{11}O_8S^- + H_2O \rightarrow$ $10 <C_4H_7O_3> + 17\ C_3H_7O_5S^-$ $+ 11\ CO_2$
**Substrate assimilation** [mol/g dry mass]	6.9	16.6
**Total substrate used** [µmol]	44	117
**Assimilated substrate** [µmol]	10.0	18.5
**Dissimilated substrate** [µmol]	34.0	98.5
**Succinate produced** [µmol]	6	12
**Formate produced** [µmol]	58	44
**Acetate produced** [µmol]	31	51
**Ethanol produced** [µmol]	11	0
**DHPS produced** [µmol]	0	96
**dissimilation equation**	34.0 glucose $\rightarrow$ 6 succinate + 58 formiate + 31 acetate + 11 ethanol	98.5 SQ $\rightarrow$ 95.9 DHPS + 12 succinate + 44 formiate + 51 acetate
**total carbon in dissimilated substrate** [µmol]	204.1	591.2
**total carbon in fermentation products** [µmol]	166.0	482.0
**Carbon recovery** [%]	**81**	**82**
**Molar growth yield for dissimilated substrate** [g dry mass/mol diss. substrate]	**42.6**	**11.3**
**Electrons gained by carbon oxidation**	1 glucose $\rightarrow 6\ CO_2 + 24\ e^-$ 1 SQ $\rightarrow 6\ CO_2 + 24\ e^-$	1 formate $\rightarrow 1\ CO_2 + 2\ e^-$ 1 ethanol $\rightarrow 2\ CO_2 + 12\ e^-$ 1 DHPS $\rightarrow 3\ CO_2 + 14\ e^-$
**Electrons in dissimilated substrate**	816	2365
**Electrons in fermentation products**	580	2008
**Electron recovery** [%]	**71**	**85**

Table S2: PCR primers used for cloning

Enzyme	Gene (IMG locus tag)	forward Primer (restriction: NdeI)	reverse primer (restriction: XhoI)
DHPS dehydrogenase	Ga0134130_130620	*CGT***CATATG**CAGATCGGATTTATCGGC	*ATT***CTCGAG**ATCCGGCTCTTCCACCTCAA
SLA dehydrogenase	Ga0134130_130623	*CGT***CATATG**TTGACAACATTCGAACTCATGCG	*ATT***CTCGAG**GGTTGCCCTATCCTTATCCGTTTTG
SuyA	Ga0134130_10402	*CGT***CATATG**TCTATCCAATTTATTGTCCACGAA	*ATT***CTCGAG**GTCCATTCTCACGCCGATACC
SuyB	Ga0134130_10403	*CGT***CATATG**AAGACCAAGTTCATGGGGTATCG	*ATT***CTCGAG**AACCGCCGGCCCGACTAC

Letters in bold, restriction enzyme recognition site;
letters in italic, base pairs added for restriction efficiency.

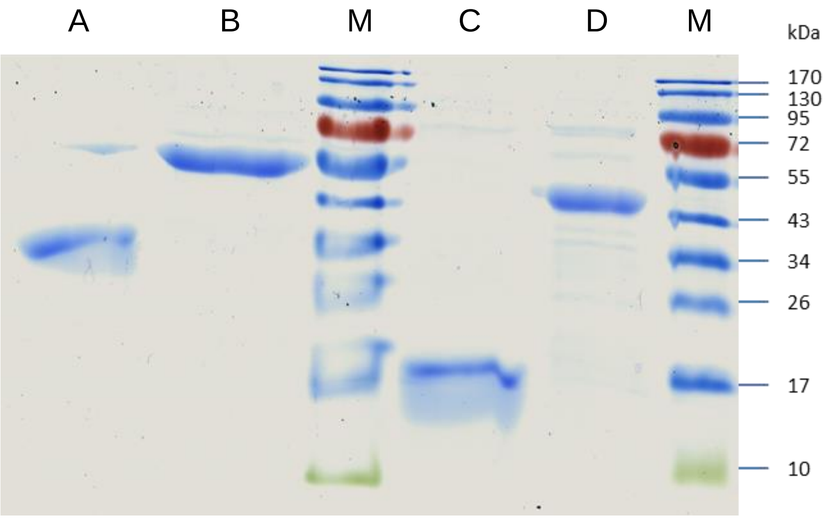

Fig S1: Evaluation of the purity of recombinant His-tagged proteins by SDS-PAGE
(A) DHPS dehydrogenase (locus tag Ga0134130_130620, calculated mass 33.3 kDa);
(B) SLA dehydrogenase (Ga0134130_130623, 54.9 kDa);
(C) SuyA (Ga0134130_10402, 14.5 kDa);
(D) SuyB (Ga0134130_10403, 45.9 kDa);
(M) molecular mass marker.
